# Hemoadsorption as a Supportive Strategy for Severe Toxicity Associated With Chimeric Antigen Receptor T-Cell Therapy: A Case Series

**DOI:** 10.1016/j.xkme.2025.101001

**Published:** 2025-04-03

**Authors:** Pasquale Esposito, Massimiliano Gambella, Elisa Russo, Anna Maria Raiola, Elena Beltrametti, Novella Conti, Elisa Porcile, Stefania Bianzina, Monica Centanaro, Francesca Viazzi, Emanuele Angelucci

**Affiliations:** 1 Department of Internal Medicine (DIMI), University of Genova, Genova, Italy; 2 Unit of Nephrology, Dialysis and Transplantation, IRCCS Ospedale Policlinico San Martino, Genova, Italy; 3 Unit of Hematology and Cellular Therapy, IRCCS Ospedale Policlinico San Martino, Genova, Italy; 4 Anesthesiology and Intensive Care Unit, IRCCS Ospedale Policlinico San Martino, Genova, Italy; 5 Neonatal and Pediatric Intensive Care Unit, Emergency Department, IRCCS Istituto Giannina Gaslini, Genova, Italy

**Keywords:** B-cell lymphoma, blood purification, CAR-T therapy, cytokine release syndrome, hemoadsorption, interleukin-6

## Abstract

**Rationale & Objective:**

To describe the use and effects of extracorporeal blood purification with hemoadsorption in managing severe complications after treatment with chimeric antigen receptor T-cells (CAR-T).

**Study Design:**

Retrospective analysis of a case series.

**Setting & Participants:**

Hematological department and intensive care unit from a single institution. Patients with hematological cancer who underwent CAR-T therapy between 2021 and 2023, developed severe toxicity, and were treated with hemoadsorption based on clinical indications.

**Results:**

Of 48 patients, extracorporeal blood purification was prescribed to 4 (8.3%): 3 with diffuse large B-cell lymphoma and 1 with mantle cell lymphoma. These patients experienced rapid increases in serum interleukin-6 and ferritin levels after CAR-T infusion, which progressed to severe cytokine release syndrome with hemodynamic instability and multiple-organ toxicity. Despite corticosteroid and anakinra rescue therapy after tocilizumab failure, extracorporeal blood purification with hemoadsorption was initiated at a mean of 5.2 ± 1.7 days following CAR-T infusion due to rapid clinical deterioration. The treatment was performed using continuous venovenous hemodiafiltration with an AN69ST hemofilter and a CytoSorb cartridge. One patient died 1 day after the initiation of blood purification because of concomitant cardiomyopathy progressing to multiple-organ failure. In the 3 surviving patients, interleukin-6 levels significantly decreased (from −18% to −95%), cytokine release syndrome resolved, and vasoactive support was reduced. Treatment-related complications were not observed.

**Limitations:**

Small sample size, retrospective design, and lack of a predefined hemoadsorption therapy protocol.

**Conclusions:**

A strategy based on hemoadsorption was safe and effective in mitigating inflammation and improving hemodynamics in patients with hematological cancer treated with CAR-T therapy who developed early severe toxicity.

## Plain Language Summary

Chimeric antigen receptor T-cell (CAR-T) therapy may improve remission rates and survival in hematological cancers but can lead to severe side effects, including cytokine release syndrome (CRS). CRS is characterized by systemic inflammation and multiorgan toxicity and is often associated with poor outcomes. Although pharmacological treatments are available, some cases remain refractory. In this study, we share our experience with hemoadsorption as a supportive therapy for severe CRS in CAR-T recipients. Of the 4 patients treated, 3 experienced CRS resolution with reduced inflammation and improved hemodynamic stability, without treatment-related complications. These findings highlight hemoadsorption as a promising adjunctive therapy to pharmacological treatments for managing severe CAR-T toxicities. Further research is needed to confirm these results.

Chimeric antigen receptor T-cell (CAR-T) therapy, a novel approach for the treatment of hematological malignancies, involves the use of genetically engineered T cells that target and destroy cancer cells.^1^ The process includes collecting a patient’s cells, modifying them to express CARs that recognize cancer-specific antigens, and then reinfusing them into the patient.^2^ Current indications for CAR-T treatment include relapsed or refractory B-cell leukemia, lymphoma, and multiple myeloma; this treatment modality can potentially lead to disease remission and increased overall and disease-free survival.^3^ However, CAR-T therapy poses risks of side effects, such as immune effector cell-associated neurotoxicity syndrome (ICANS), hematological and cardiac toxicity, and acute kidney injury (AKI).^4^ The most prevalent and significant complication is cytokine release syndrome (CRS), which can occur in a high proportion of patients undergoing CAR-T treatment (20%-90%).^5^

CRS is a systemic inflammatory response triggered by the rapid release of a large amount of proinflammatory cytokines, such as interleukin-6 (IL-6), induced by CAR-T treatment.^6^ Symptoms of CRS range from mild flu-like symptoms to life-threatening multiple-organ dysfunction, presenting with symptoms such as fever, severe hypotension, cardiomyopathy, acute respiratory distress syndrome, and capillary leak syndrome.^7^

The management of CRS involves supportive care and administration of anti-inflammatory agents, such as tocilizumab, corticosteroids, and anakinra.^8^ Extracorporeal blood purification techniques, including hemoadsorption, have primarily been used for septic shock to remove excess inflammatory molecules and cytokines.^9^ In anecdotal cases, this technique has also been explored for severe or refractory CRS following CAR-T therapy, yielding promising results.^10^

This case series reports our experience on the efficacy of hemoadsorption as a supportive strategy for patients with severe multiple-organ toxicity associated with CAR-T therapy.

## Patients and Methods

We retrospectively analyzed data from patients who underwent CAR-T therapy for hematological indications at our center between 2020 and 2023. In this cohort, we identified patients treated with hemoadsorption for clinical indications. For these patients, we collected demographic data, comorbid condition and hematological history, details of CAR-T infusion, and subsequent follow-up data, including the onset of complications and related treatments.

The inflammatory pattern was evaluated by measuring serum IL-6, ferritin, and C-reactive protein levels using standard laboratory techniques. Additionally, we gathered data regarding hemoadsorption to assess changes in clinical and inflammatory parameters during extracorporeal treatment. Patient outcomes included CRS resolution, disease progression, and death.

The Ann Arbor staging system was used for the anatomic staging of disease.^11^ CRS was graded according to the American Society for Transplantation and Cellular Therapy criteria based on the degree of fever, hypoxia, and hypotension.^12^ ICANS was graded according to the severity of neurological symptoms, including cognitive dysfunction, level of consciousness, motor weakness, seizures, and cerebral edema, as per the American Society for Transplantation and Cellular Therapy guidelines.^12^

AKI was defined according to the Kidney Disease: Improving Global Outcomes (KDIGO) criteria,^13^ considering the increases in serum creatinine levels compared with pre–CAR-T infusion values, and decreased urine output. AKI was subsequently classified into 3 stages: stage 1 (1.5-1.9 times baseline creatinine), stage 2 (2-2.9 times baseline creatinine), and stage 3 (≥3 times baseline creatinine). The Eastern Cooperative Oncology Group performance status was used to evaluate patients’ functional statuses.^14^

Finally, the mantle cell lymphoma international prognostic index and international prognostic index were used to stratify patients based on the severity and prognosis of mantle cell lymphoma and diffuse large B-cell lymphoma, respectively.^15^

The study protocol was approved by our institutional review board (registration number: 515/2020), and the requirement for informed consent was waived. This study adhered to the principles outlined in the Declaration of Helsinki.

## Case Series

Of 48 patients (29 men and 19 women; mean age 61 ± 11 years) who received CAR-T therapy during the study period, 4 patients (8.3%) were treated with hemoadsorption (Table 1): 3 with diffuse large B-cell lymphoma and 1 with mantle cell lymphoma. All patients had a history of refractory B-cell lymphoma treated with multiple therapeutic lines. At the time of CAR-T infusion, they presented with a high disease burden and extranodal involvement. Additionally, all patients had a low performance status and a high-risk profile. Before CAR-T infusion, all patients underwent lymphodepletion with fludarabine and cyclophosphamide for 3 days. The 3 patients with diffuse large B-cell lymphoma underwent infusion with the tisagenlecleucel CAR-T product, whereas the patient with mantle cell lymphoma received brexucabtagene autoleucel (Table 2).

**Table 1:** Main Clinical and Laboratory Characteristics at Baseline (Day of CAR-T Infusion) in Patients Treated With Hemoadsorption

Patient	1	2	3	4
Clinical features				
Age, y	69	47	77	75
Sex	F	F	M	F
Hypertension	No	No	Yes	Yes
Diabetes	No	No	Yes	No
Charlson Index	4	2	6	6
Hematological Diagnosis	NOS-DLBCL	NOS-DLBCL	MCL	NOS-DLBCL
previous therapy lines	2	2	3	2
disease stage	4B	4A	3SB	4B
IPI/MIPI	High	High	High	High
ECOG	3	4	3	3
Laboratory				
LDH, U/L	992	3,059	470	647
CRP, mg/L	40	59.3	54.7	16.4
IL-6, ng/L	21	11	41	48
Ferritin, μg/L	322	3,131	1,599	450
Platelets, ×10^9^/L	56	51	19	168
sCr, mg/dL	0.6	1.5	0.7	0.7

Abbreviations: CAR-T, chimeric antigen receptor T-cell; CRP, C-reactive protein; ECOG, Eastern Cooperative Oncology Group; F, female; IL-6, interleukin-6; IPI, international prognostic index; LDH, lactate dehydrogenase; M, male; MCL, mantle cell lymphoma; MIPI, mantle cell lymphoma international prognostic index; NOS-DLBCL, diffuse large B-cell lymphoma, not otherwise specified; sCr, serum creatinine.

**Table 2:** CAR-T Therapy Schedule, Postinfusion Complications, and Management in Patients Treated With Hemoadsorption

Patient	1	2	3	4
CAR-T therapy				
Lymphodepletion regimen	Fluda 25 mg Cy 250 mg	Fluda 25 mg Cy 250 mg	Fluda 30 mg Cy 500 mg	Fluda 25 mg Cy 250 mg
CAR-T product	Tisagenlecleucel	Tisagenlecleucel	Brexucabtagene autoleucel	Tisagenlecleucel
Postinfusion complications				
CRS	Yes	Yes	Yes	Yes
Onset from infusion	0	1	2	1
Max stage	4	4	4	4
ICANS	Yes	Yes	Not valuable	Yes
Onset from infusion	5	2	—	1
Max stage	4	3	—	4
Other toxicities			Coagulopathy	Cardiomyopathy
ICU admission from infusion, d	3	3	4	1
Vasoactive support	Yes	Yes	Yes	Yes
IMV	Yes	Yes	Yes	Yes
Peak IL-6, ng/L	>5,000^a^	4,842	4,575	2,974
Day from infusion	3	3	5	4
Peak CRP, mg/L	148	221	82	46.6
Day from infusion	3	3	2	2
Peak ferritin, μg/L	6,564	50,565	29,330	56,401
Day from infusion	10	5	8	5
Peak sCr, mg/dL	1.9	2.4	1.7	1.5
Day from infusion	7	3	5	5
AKI stage	3	3 (with oliguria)	2	3
Pharmacological treatment				
Tocilizumab	Yes	Yes	Yes	Yes
First dose (day from CAR-T)	2	1	2	1
Cumulative dose, mg	1,920	1,920	1,500	1,600
Corticosteroids (dexamethasone)	Yes	Yes	Yes	Yes
First dose (day from CAR-T)	4	2	4	2
Cumulative dose, mg	170	540	790	270
Anakinra	Yes	Yes	Yes	Yes
First dose (day from CAR-T)	7	2	4	3
Cumulative dose, mg	700	1,300	700	1,400

Abbreviations: AKI, acute kidney injury; CAR-T, chimeric antigen receptor T-cell; CRP, C-reactive protein; Cy, cyclophosphamide; CRS, cytokine release syndrome; Fluda, fludarabine; ICANS, immune effector cell-associated neurotoxicity syndrome; ICU, intensive care unit; IL-6, interleukin-6; IMV, invasive mechanical ventilation; sCr, serum creatinine.

a Above laboratory limit.

All patients developed early and severe grade 4 CRS, characterized by hyperpyrexia, rapid clinical deterioration, and markedly elevated levels of inflammatory markers. Severe ICANS occurred concomitantly in 3 patients. All patients required intensive care with vasoactive support for cardiovascular shock and mechanical ventilation for acute respiratory failure. Moreover, all patients developed AKI; urinary output was preserved in 3 patients, while oliguria was observed in 1 patient.

According to the current clinical practice and recommendations, patients underwent standard CRS management, including administration of tocilizumab, corticosteroids, and anakinra. However, because this multitarget pharmacological approach did not result in prompt improvement of the hyperinflammatory syndrome, the therapy was intensified by incorporating extracorporeal blood purification coupled with hemoadsorption.

The combined treatment was initiated after a mean of 5.2 ± 1.7 days after CAR-T infusion. All patients underwent continuous venovenous hemodiafiltration using the CytoSorb adsorbing cartridge (CytoSorbents Corporation), incorporated in series with an AN69ST membrane hemofilter on a continuous renal replacement therapy platform (Prismaflex; Baxter) with initial blood flow rates of 100 to 150 mL/min. Each adsorbent cartridge was used for 24 hours with repeated treatments based on the clinical indications.

After these treatments, standard continuous venovenous hemodiafiltration was continued for each patient as long as clinically indicated. Notably, no treatment-related complications were observed; thrombocytopenia, which was partially present before CAR-T therapy, was attributed to lymphodepletion chemotherapy and concurrent inflammation.

Hemoadsorption was associated with a significant reduction in IL-6 (from −18% to −95%) and ferritin levels (from −29% to −67%). Patient 4 (Table 3), who exhibited a less pronounced reduction in IL-6 levels, developed concomitant severe cardiomyopathy. This patient died after only 1 cycle of hemoadsorption owing to severe multiple-organ dysfunction syndrome.

**Table 3:** Operative Parameters and Laboratory and Clinical Characteristics of CAR-T Patients Treated With Hemoadsorption-Based Blood Purification

Patient	Days From CAR-T Infusion	Blood Purification Modality	Dialysis Membranes	Anticoagulation	No. HA cycles	CVVHDF Duration (d)	IL-6 at HA Start (ng/L)	IL-6 at HA Stop (ng/L)	Delta IL-6 (%)	NE at HA Start (μg/kg/min)	NE at HA Stop (μg/kg/min)	CRS Resolution
1	7	CVVHDF	modified AN69S + *CytoSorb*	RCA	2	6	>5,000^a^	255	−95%	0.03	Stop	Yes
2	3	CVVHDF	AN69ST + *CytoSorb*	RCA	3	11	4,842	559	−88%	0.4	Stop	Yes
3	6	CVVHDF	modified AN69S + *CytoSorb*	RCA	4	5	4,575	1,477	−67%	0.14	Stop	Yes
4	5	CVVHDF	AN69ST + *CytoSorb*	No	1	1	2,452	1,997	−18%	0.5	0.7	No^b^

Abbreviations: CAR-T, chimeric antigen receptor T-cell; CRS, cytokine release syndrome; CVVHDF, continuous venovenous hemodiafiltration; HA, hemoadsorption; IL-6, interleukin-6; NE, norepinephrine; RCA, regional citrate anticoagulation.

a Above laboratory limit.

b Patient with concomitant severe cardiomyopathy.

In contrast, the remaining 3 patients, in whom other causes of shock were excluded, achieved complete CRS resolution (Table 3). Notably, these patients experienced improvements in their hemodynamic conditions, allowing gradual reduction and discontinuation of vasoactive support (Tables S1-S4 and Figures S1-S4). Respiratory mechanics and gas exchange also improved, and 2 patients were weaned from mechanical ventilation. Moreover, all patients experienced full recovery of kidney function.

Of the 3 patients who survived CRS, 2 died of disease progression and infection. Only 1 patient survived, who ultimately died due to disease progression 2 months after hospital discharge.

## Discussion

Herein, we report our experience of using hemoadsorption to treat CRS associated with CAR-T therapy. In patients with severe CRS and multisystemic involvement, combining hemoadsorption with conventional blood purification therapy led to significant reductions in inflammatory markers and clinical improvements, especially regarding hemodynamic stability.

CRS, a common and potentially life-threatening complication of CAR-T therapy, is primarily triggered by the release of inflammatory factors from activated immune and endothelial cells.^16^ Risk factors for severe CRS include cancer-related aspects such as high tumor burden and poor performance status and treatment-related factors such as the type of costimulatory domain and the use of fludarabine.^7^^,^^16^ Our patients were at a high risk due to advanced hematological neoplasms and comorbid conditions, such as diabetes and hypertension, contributing to a poor prognosis.

The course of CRS can be further complicated by multiple-organ toxicities, including ICANS, AKI, acute respiratory distress syndrome, cardiovascular and liver failure, and cytopenia.^17^^,^^18^ The pharmacological treatment of CRS includes a multitarget approach. Tocilizumab, an IL-6 receptor antagonist, is the first-line treatment for IL-6-mediated hyperinflammation^19^; if ineffective, corticosteroids can be initiated. For severe, unresponsive CRS, additional agents, such as anakinra and JAK 1-2 inhibitors may be used.^20^ In our cases, these strategies were used sequentially without any evident benefits, prompting the use of hemoadsorption.

Hemoadsorption is based on the interaction between blood and an adsorbent material that can remove circulating toxins through mechanisms such as hydrophobic interactions and hydrogen bonding.^21^ Some evidence suggests that hemoadsorption may be effective in reducing cytokine levels and improving outcomes in patients with CRS of different etiologies, including sepsis, as well as noninfectious causes such as CRS following immunotherapy or cardiac surgery.^22^^,^^23^

In our cases, we used the CytoSorb cartridge, which consists of highly biocompatible porous polymer beads that are capable of extracting molecules up to a size of 65 kDa.^24^ Originally developed for the management of septic shock,^25^ CytoSorb has since been applied across a spectrum of conditions, including necrotizing fasciitis, meningococcal sepsis, pancreatitis, trauma, hyperbilirubinemia, rhabdomyolysis, poisoning, and, more recently, COVID-19-related CRS.^26^^,^^27^

Nevertheless, the clinical benefits of hemoadsorption in these contexts remain largely limited to anecdotal reports, small case series, and a few small randomized trials, and its impact on mortality remains uncertain.^28^, ^29^, ^30^ Similarly, only isolated reports have suggested the potential utility of hemoadsorption in mitigating CAR-T-related CRS and ICANS by reducing proinflammatory cytokine levels.^10^^,^^31^^,^^32^

Our findings contribute to the growing body of evidence supporting the potential of hemoadsorption as an adjunctive treatment, together with pharmacological therapies, for severe and refractory CRS. However, despite the relevant role of hemoadsorption in resolving inflammation and improving organ dysfunction, our patients had poor outcomes, primarily because of underlying disease progression. This highlights the complexity of such clinical conditions and the multiple risk factors present in these patients before CAR-T infusion.

Our study has limitations owing to its descriptive and retrospective nature and the lack of a predefined protocol for hemoadsorption therapy in this specific clinical setting. In addition, distinguishing between the effects of pharmacological therapy and those of extracorporeal removal is challenging. Furthermore, the absence of a control group may make it difficult to determine the true efficacy of concurrent application of hemoadsorption with standard treatments. Additionally, access to hemoadsorption is not universal across intensive care units, and the high cost may limit its availability. This could pose a significant barrier to the widespread adoption and generalization of this approach.

Nevertheless, our results are important from several perspectives. First, they emphasized the need for initial risk stratification to guide the management of patients treated with CAR-T therapy. Thus, the potential efficacy of extracorporeal treatment in this context could expand the indications for hemoadsorption therapy as a supportive strategy for managing severe systemic complications of CAR-T therapy. This is relevant as CAR-T therapy is expected to evolve, with its indications expanding to various tumor types and non-tumor conditions, such as autoimmune diseases.^33^

In conclusion, while awaiting results from ongoing randomized trials,^34^ our data suggest that a strategy based on hemoadsorption may offer an additional therapeutic option for mitigating CAR-T-related CRS in selected patients with refractory diseases. Larger controlled studies are needed to validate these preliminary findings and define the patient selection criteria, timing, treatment schedule, and concomitant anti-inflammatory therapies.
