# Supplementary material for: Hemoadsorption as a Supportive Strategy for Severe Toxicity Associated With Chimeric Antigen Receptor T-Cell Therapy: A Case Series
Source: Kidney Med. 2025 Apr 3;7(6):101001. doi: 10.1016/j.xkme.2025.101001 (PMC12152617; doi:10.1016/j.xkme.2025.101001)
Supplement: Supplementary File (PDF) — Figures S1-S4; Tables S1-S4. [file mmc1.pdf]

ESPOSITO, KIDNEY MED, “HEMOADSORPTION AS A SUPPORTIVE STRATEGY FOR SEVERE TOXICITY ASSOCIATED WITH CHIMERIC ANTIGEN RECEPTOR T CELL THERAPY: A CASE SERIES”

Supplementary Tables

Clinical and laboratory course of CAR-T patients treated with hemoadsorption

Table S1. Patient #1

Days	CAR-T infusion	2	3	5	7	8	9	10	12	15	19	23	Outcome
Day on CVVHDF					X	X	X	X	X				Death day 41 (disease progression)
Day on HA					X	X							
WBC (x µl)	560	630	360	140	1850	3820	3600	4000	3700	1440	1500	600	
PLT (x10^9/L)	56	32	26	17	16	17	14	20	10	13	16	52	
IL-6 (ng/L)	20.6	419	>5000*	>5000*	>5000*	713	355	112	255	111	312	2159	
CRP (mg/L)	40.2	79.5	148	25.1	24.6	15.6	10.2	5.7	2	2	2	2	
LDH (U/L)	922	623	579	706	866	841	871	805	645	638	1240	1731	
Ferritin (µg/L)	322		373	1772	5926			6564	4239	1933	1581	1719	
sCr (mg/dl)	0.6	0.6	0.5	0.7	1.9	1.6	1.4	1.1	0.9	0.7	0.5	0.6	
CRS stage	0	1	3	3	4	4	4	4	2	0	0	0	
MAP (mmHg)	80	70.7	53	85.3	75	83.7	90	106.7	100	101	93	98	
NE dose (µg/kg/min)	-	-	0.03	0.03	0.12	0.06	0.03	Stop					
Respiratory support	-	LFO	LFO	LFO	CPAP	IMV	IMV	IMV	IMV-> LFO	LFO	LFO	LFO	
P/F (mmHg)			184	167	135	143	184	160	341	269	300	325	

Abbreviations: Chimeric Antigen Receptor T-cell (CAR-T), Continuous Veno-Venous Hemodiafiltration (CVVHDF), Hemoadsorption (HA), WBC (white blood cells), PLT (platelets), Interleukin-6 (IL-6), C-Reactive Protein (CRP), Lactate Dehydrogenase (LDH), serum Creatinine (sCr), Cytokine Release Syndrome (CRS), Mean Arterial Pressure (MAP), Norepinephrine (NE), Low-Flow Oxygen therapy (LFO), Continuous Positive Airway Pressure (CPAP), Invasive Mechanical Ventilation (IMV), PaO_2/FiO_2 ratio (P/F).
*Above laboratory limit

Table S2. Patient #2

Days	CAR-T infusion	2	3	4	5	7	8	9	10	12	15	19	23	Outcome
Day on CVVHDF			X	X	X	X	X	X	X	X				Discharge day 49
Day on HA			X	X	X									
WBC (x µl)	9840	2170	920	270	1650	8360	6270	4470	4200	5740	3240	4900	3350	
PLT (x10^9/L)	51	19	19	15	44	35	14	16	22	15	11	17	14	
IL-6 (ng/L)	11,1	1693	>5000*	>5000*	4842	4551	2648	1705	1192	559	458	276	123	
CRP (mg/L)	59,3	158	221	129	46,1	21,9	15	12.8	11.3	13	13	5.8	2	
LDH (U/L)	3059	1943	751	933	1241	1890	169	1274	974	834	800	421	391	
Ferritin (µg/L)	3162	5219	12706	45297	50565	32960	27088	22202	9874	8354	5027	2761	2200	
sCr (mg/dl)	1.4	1.7	2.4	1.8	1.2	1.2	1.1	1	1.2	0.8	1	0.7	0.7	
CRS stage	0	2->3	4	4	4	4	4	3	2 o 3	1	0	0	0	
MAP (mmHg)	96.7	74	65	53	73	85	90	92	106.7	105.7	93	84	92	
NE dose (µg/kg/min)	-	0.08	0.4	0.3	0.3	0.05	0.03	Stop						
Respiratory support	-	LFO	NIV->IMV	IMV	IMV	IMV	IMV	IMV	IMV	IMV	IMV	CPAP	LFO	
P/F (mmHg)		322	157	197	287	285	203	189	297	450	397	493	469	

Abbreviations: Chimeric Antigen Receptor T-cell (CAR-T), Continuous Veno-Venous Hemodiafiltration (CVVHDF), Hemoadsorption (HA), WBC (white blood cells), PLT (platelets), Interleukin-6 (IL-6), C-Reactive Protein (CRP), Lactate Dehydrogenase (LDH), serum Creatinine (sCr), Cytokine Release Syndrome (CRS), Mean Arterial Pressure (MAP), Norepinephrine (NE), Low-Flow Oxygen therapy (LFO), Non Invasive Ventilation (NIV), Invasive Mechanical Ventilation (IMV), Continuous Positive Airway Pressure (CPAP), PaO_2/FiO_2 ratio (P/F).
*Above laboratory limit

Table S3. Patient #3

Days	CAR-T infusion	2	3	5	6	7	8	9	10	12	14	15	Outcome
Day on CVVHDF					X	X	X	X	X	X			Death day 32 (fungal sepsis)
Day on HA					X	X	X	X					
WBC (x µl)	1570	340	120	130	290	1000	5280	4990	1100	920		890	
PLT (x10^9/L)	19	22	14	27	11	6	8	7	11	14		40	
IL-6 (ng/L)	41.4	1568		4575	>5000*	>5000*	1851	896	756	661	722	1165	
CRP (mg/L)	54.7	82	31	31	22.6	24.5	19	14	7.3	3	3.3	5.9	
LDH (U/L)	470	449	495	536	639	110	132	741	492	346	401	410	
Ferritin (µg/L)	1599	1943		6699		23853	29330	20083	12427	8040	6785	5004	
sCr (mg/dl)	0.7	0.8	0.8	1.7	1	0.8	1.1	1.2	0.9	0.9	1.1	1.2	
CRS stage	0	1	1	3	4	4	4	4	3	2	0	0	
MAP (mmHg)	92.7	92	87	47	53	83	93	88	73.7	88	78	97	
NE dose (µg/kg/min)	-	-	-	0.12	0.18	0.4	0.33	0.05	Stop				
Respiratory support	-	-	LFO	LFO-> IMV	IMV	IMV	IMV	IMV	IMV	IMV	IMV	IMV	
P/F (mmHg)	400			292	241	277	300	206	163	320	286	265	

Abbreviations: Chimeric Antigen Receptor T-cell (CAR-T), Continuous Veno-Venous Hemodiafiltration (CVVHDF), Hemoadsorption (HA), WBC (white blood cells), PLT (platelets), Interleukin-6 (IL-6), C-Reactive Protein (CRP), Lactate Dehydrogenase (LDH), serum Creatinine (sCr), Cytokine Release Syndrome (CRS), Mean Arterial Pressure (MAP), Norepinephrine (NE), Low-Flow Oxygen therapy (LFO), Invasive Mechanical Ventilation (IMV), PaO_2/FiO_2 ratio (P/F).
*Above laboratory limit

Table S4. Patient #4

Days	CAR-T infusion	2	3	5	6	Outcome
Day on CVVHDF				X	X	Death day 6 (myocardial toxicity)
Day on HA				X		
WBC (x µl)	1240	340	320	6490	9430	
PLT ($x10^9$/L)	168	121	91	176	199	
IL-6 (ng/L)	48	1331	2174	2454	1997	
CRP (mg/L)	54.7	82	31	31	22.6	
LDH (U/L)	647	483	516	1357	4000	
Ferritin (µg/L)	450	526	541	56401	40000	
sCr (mg/dl)	0.7	0.7	0.8	1.5	1.7	
CRS stage	0-1	2	3	2	4	
MAP (mmHg)	85	86.7	50	87	37	
NE dose (µg/kg/min)	-	-	0.05	0.2	0.7	
Respiratory support	-	LFO	LFO	LFO-> IMV	IMV	
P/F (mmHg)		425	332	440	151	

Abbreviations: Chimeric Antigen Receptor T-cell (CAR-T), Continuous Veno-Venous Hemodiafiltration (CVVHDF), Hemoadsorption (HA), WBC (white blood cells), PLT (platelets), Interleukin-6 (IL-6), C-Reactive Protein (CRP), Lactate Dehydrogenase (LDH), serum Creatinine (sCr), Cytokine Release Syndrome (CRS), Mean Arterial Pressure (MAP), Norepinephrine (NE), Low-Flow Oxygen therapy (LFO), Invasive Mechanical Ventilation (IMV), PaO_2/FiO_2 ratio (P/F).

Supplementary Figures

Individual clinical course and management of CAR-T patients treated with hemoadsorption.

Figure S1

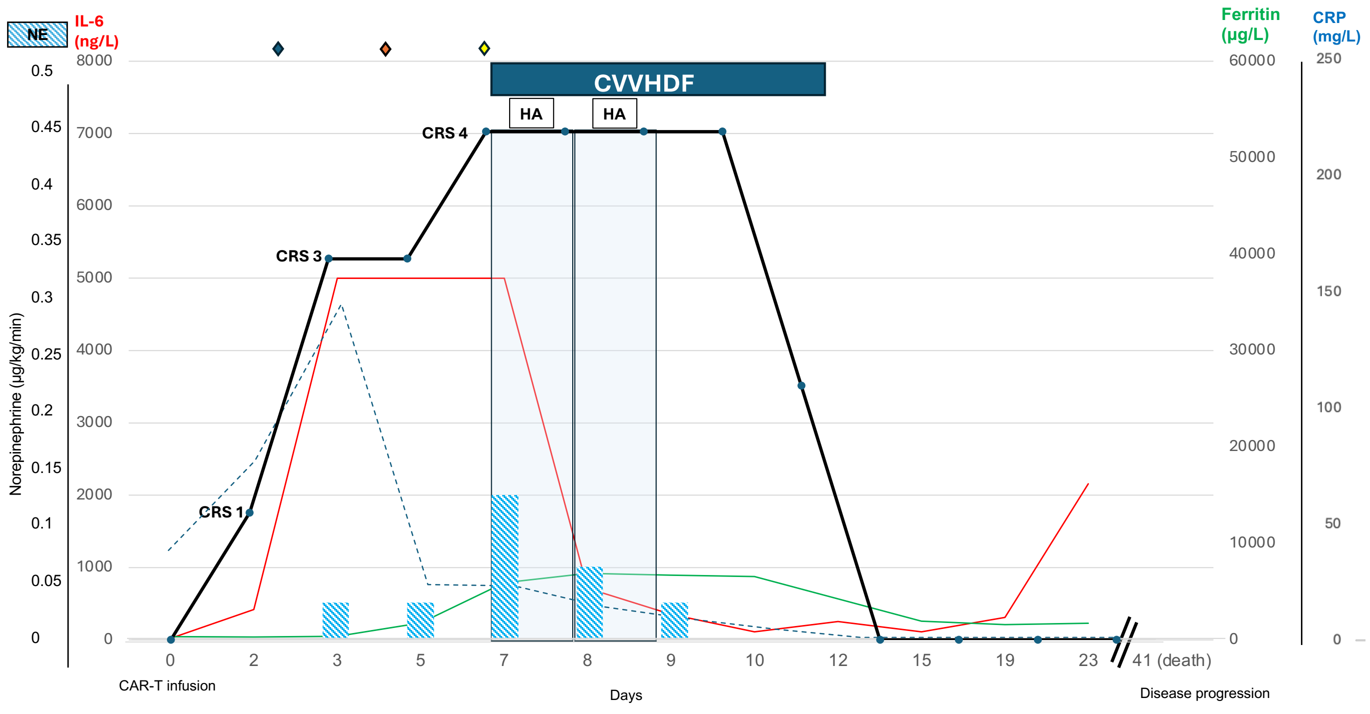

Patient #1

Black line= CRS grading, Red line= IL-6 levels, Green line= Ferritin levels, Blu line= CRP levels,

Light blue columns denote the NE dose. Light blue columns depict the NE dose. Treatment initiation dates are marked by colored rhombuses: black for tocilizumab, red for corticosteroids, and yellow for anakinra. Each hemoadsorption cycle is indicated by an "HA" box.

Abbreviations: Chimeric Antigen Receptor T-cell (CAR-T), Hemoadsorption (HA), Continuous Veno-Venous Hemodiafiltration (CVVHDF), Norepinephrine (NE), Interleukin-6 (IL-6), C-Reactive Protein (CRP), Cytokine Release Syndrome (CRS).
For IL-6, the level of 5000 ng/L represents the upper limit of the laboratory reference range.

Figure S2

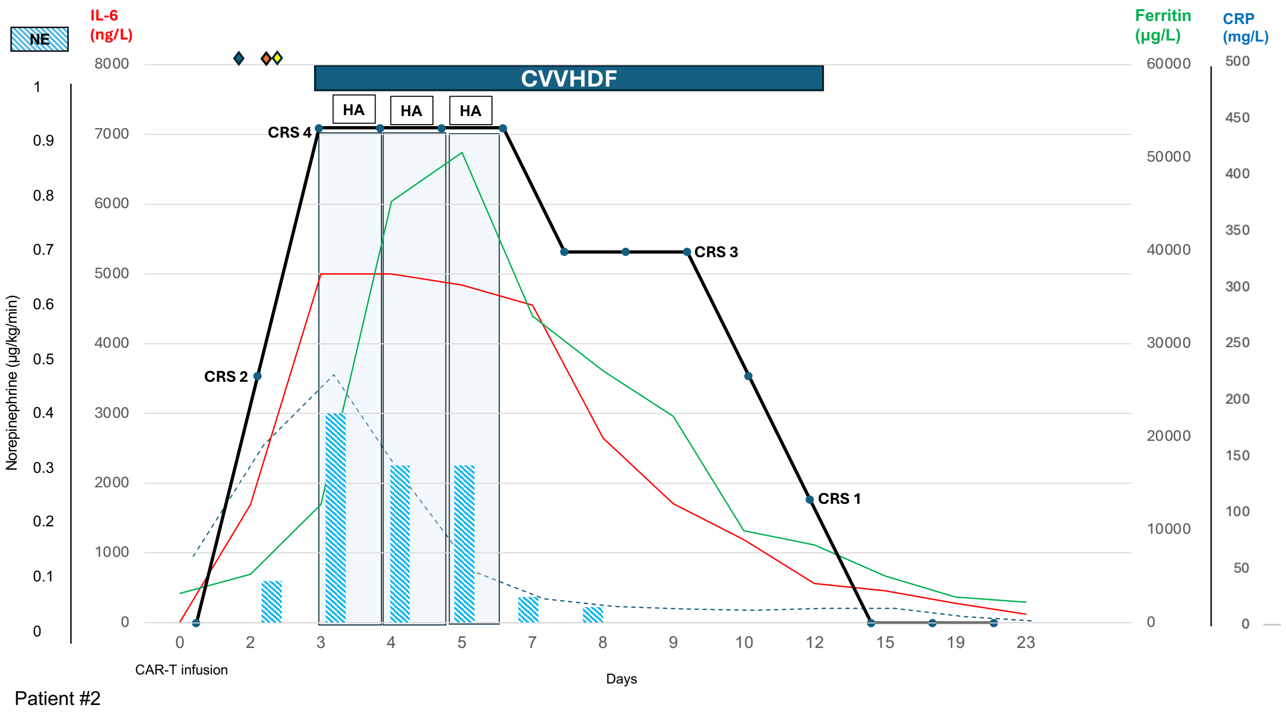

Black line= CRS grading, Red line= IL-6 levels, Green line= Ferritin levels, Blu line= CRP levels,

Light blue columns denote the NE dose. Light blue columns depict the NE dose. Treatment initiation dates are marked by colored rhombuses: black for tocilizumab, red for corticosteroids, and yellow for anakinra. Each hemoadsorption cycle is indicated by an "HA" box.

Abbreviations: Chimeric Antigen Receptor T-cell (CAR-T), Hemoadsorption (HA), Continuous Veno-Venous Hemodiafiltration (CVVHDF), Norepinephrine (NE), Interleukin-6 (IL-6), C-Reactive Protein (CRP), Cytokine Release Syndrome (CRS).
For IL-6, the level of 5000 ng/L represents the upper limit of the laboratory reference range.

Figure S3

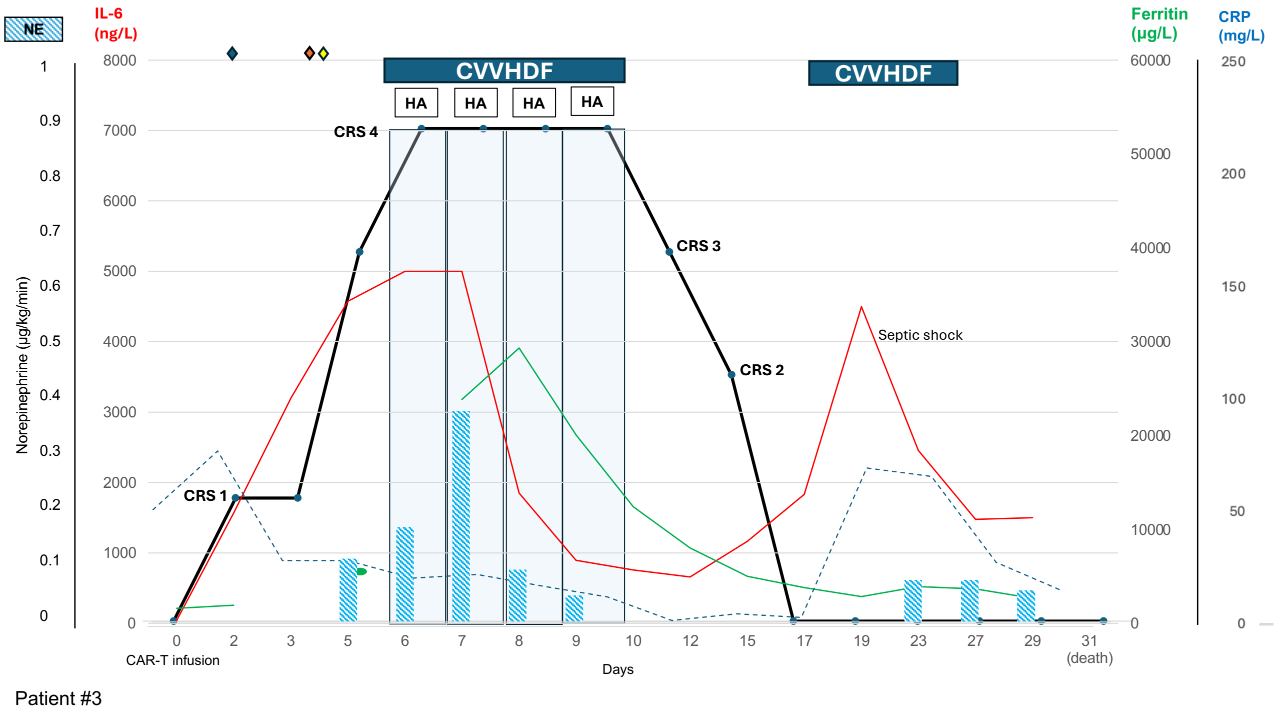

Black line= CRS grading, Red line= IL-6 levels, Green line= Ferritin levels, Blu line= CRP levels,

Light blue columns denote the NE dose. Light blue columns depict the NE dose. Treatment initiation dates are marked by colored rhombuses: black for tocilizumab, red for corticosteroids, and yellow for anakinra. Each hemoadsorption cycle is indicated by an "HA" box.

Abbreviations: Chimeric Antigen Receptor T-cell (CAR-T), Hemoadsorption (HA), Continuous Veno-Venous Hemodiafiltration (CVVHDF), Norepinephrine (NE), Interleukin-6 (IL-6), C-Reactive Protein (CRP), Cytokine Release Syndrome (CRS).

For IL-6, the level of 5000 ng/L represents the upper limit of the laboratory reference range.

Figure S4

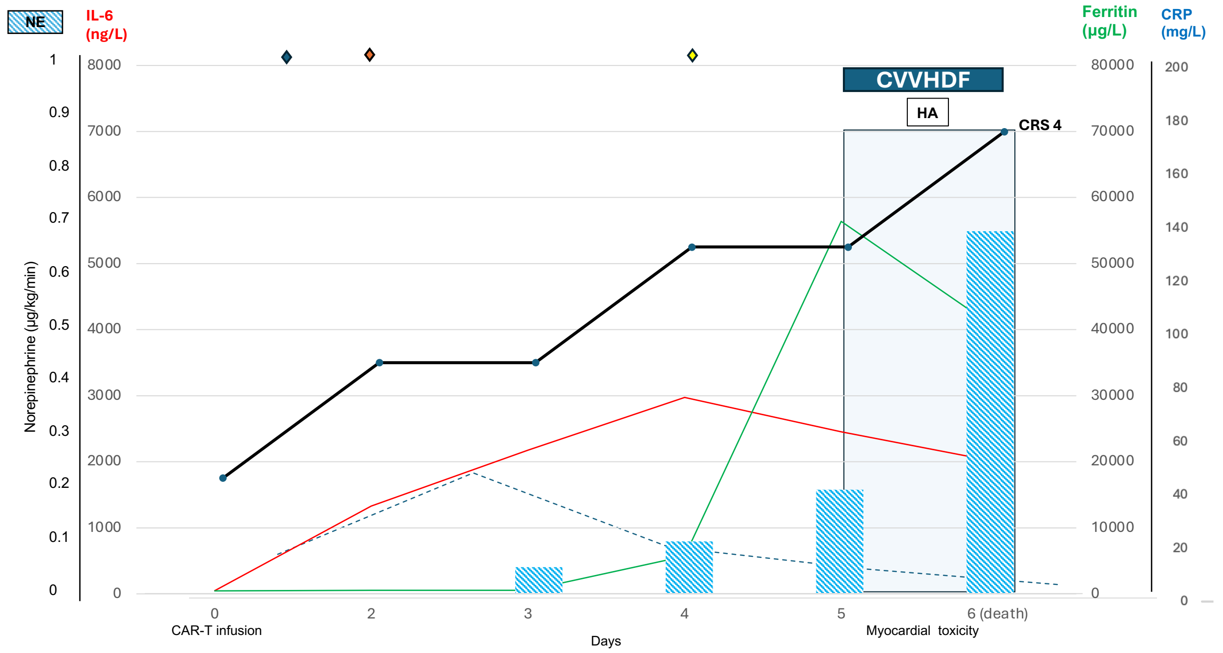

Black line= CRS grading, Red line= IL-6 levels, Green line= Ferritin levels, Blu line= CRP levels,

Light blue columns denote the NE dose. Light blue columns depict the NE dose. Treatment initiation dates are marked by colored rhombuses: black for tocilizumab, red for corticosteroids, and yellow for anakinra. Each hemoadsorption cycle is indicated by an "HA" box.

Abbreviations: Chimeric Antigen Receptor T-cell (CAR-T), Hemoadsorption (HA), Continuous Veno-Venous Hemodiafiltration (CVVHDF), Norepinephrine (NE), Interleukin-6 (IL-6), C-Reactive Protein (CRP), Cytokine Release Syndrome (CRS).
For IL-6, the level of 5000 ng/L represents the upper limit of the laboratory reference range.
